# Identification of breast cancer patients with a high risk of developing brain metastases: a single-institutional retrospective analysis

**DOI:** 10.1186/1471-2407-14-289

**Published:** 2014-04-24

**Authors:** Volker Rudat, Hamdan El-Sweilmeen, Iris Brune-Erber, Alaa Ahmad Nour, Nidal Almasri, Saleh Altuwaijri, Elias Fadel

**Affiliations:** 1 Department of Radiation Oncology, Saad Specialist Hospital, P.O. Box 30353, Al Khobar 31952, Saudi Arabia; 2 Department of Haematology and Oncology, Saad Specialist Hospital, P.O. Box 30353, Al Khobar 31952, Saudi Arabia; 3 Department of Surgery, Saad Specialist Hospital, P.O. Box 30353, Al Khobar 31952, Saudi Arabia; 4 Department of Pathology, Saad Specialist Hospital, P.O. Box 30353, Al Khobar 31952, Saudi Arabia; 5 SAAD Research & Development Center, Saad Specialist Hospital, P.O. Box 30353, Al Khobar 31952, Saudi Arabia

**Keywords:** Breast cancer, Brain metastasis, Progesterone receptor negative breast cancer

## Abstract

**Background:**

The objective of this study was to identify breast cancer patients with a high risk of developing brain metastases who may benefit from pre-emptive medical intervention.

**Methods:**

Medical records of 352 breast cancer patients with local or locoregional disease at diagnosis were retrospectively analysed. The brain metastasis-free survival was estimated using the Kaplan-Meier method and patient groups were compared using the log rank test. The simultaneous relationship of multiple prognostic factors was assessed using Cox’s proportional hazard regression analysis. The Fisher exact test was used to test the difference of proportions for statistical significance.

**Results:**

On univariate analysis, statistically highly significant unfavourable risk factors for the brain metastasis-free survival were negative ER status, negative PR status, and triple negative tumor subtype. Young age at diagnosis (≤35 years) and advanced disease stage were not statistically significant (p = 0.10). On multivariate analysis, the only independent significant factor was the ER status (negative ER status; hazard radio (95% confidence interval), 5.1 (1.8-14.6); p = 0.003). In the subgroup of 168 patients with a minimum follow-up of 24 months, 49 patients developed extracranial metastases as first metastatic event. Of those, 7 of 15 (46.6%) with a negative ER status developed brain metastases compared to 5 of 34 (14.7%) with a positive ER status (Fisher exact test, p = 0.03). The median time interval (minimum-maximum) between the diagnosis of extracranial and brain metastases was 7.5 months (1-30 months).

**Conclusions:**

Breast cancer patients with extracranial metastasis and negative ER status exhibited an almost 50% risk of developing brain metastasis during their course of disease. Future studies are highly desired to evaluate the efficacy of pre-emptive medical intervention such as prophylactic treatment or diagnostic screening for high risk breast cancer patients.

## Background

The incidence of brain metastases in breast cancer is about 5% [1,2]. While patients with early breast cancer rarely develop brain metastases, symptomatic brain metastases are diagnosed in 10% to 16% of patients with metastatic breast cancer [1,3,4]. Advances in systemic treatment have substantially improved the overall survival of advanced breast cancer patients [5,6], and brain metastases are emerging as an important sanctuary site. An increasing proportion of patients have been observed suffering from symptomatic brain metastases often at a time when their extracranial disease is apparently under control [5,7]. The survival of patients with symptomatic multiple brain metastases is poor even after palliative whole brain irradiation [8,9], and better in patients with brain oligometastases where surgical resection or stereotactic radiotherapy can be applied [10-13].

The identification of breast cancer patients with a high risk of developing brain metastases would enable pre-emptive intervention such as prophylactic treatment or diagnostic screening with the potential to improve the outcome.

Reported risk factors for brain metastases in breast cancer patients include young age at first diagnosis, presence of lung metastases, short disease-free survival, ER negative tumors, triple-negative tumor subtype, HER2 overexpression and BRCA1 phenotype [1,5,14-19].

The objective of this study was to identify a subgroup of breast cancer patients with a high risk of developing brain metastases who may benefit from pre-emptive medical intervention.

## Methods

Medical records were retrospectively reviewed of female breast cancer patients who consulted Saad Specialist Hospital between 2006 and 2013. Eligibility criteria for the analysis were histologically confirmed diagnosis of invasive breast cancer. Patients with distant metastases, synchronous, or metachronous cancer at diagnosis were excluded from the analysis. Staging procedures included complete history and physical examination, laboratory assessments, and diagnostic bilateral mammogram. Where indicated, ultrasonography of the breast and abdomen, chest radiograph, and radionuclide bone scan were performed. Selected patients received magnetic resonance imaging (MRI) of the breast, computerized tomography (CT), or positron emission tomography computed tomography (PET-CT). Patients were presented and discussed in an interdisciplinary Tumor Board Meeting, and a treatment recommendation was generated in accordance with the guidelines of the National Comprehensive Cancer Network (NCCN). Breast conserving surgery (BCS) consisted of wide local excision or lumpectomy and axillary dissection or sentinel lymph node biopsy in selected patients. After modified radical mastectomy, in selected patients breast reconstruction with TRAM-flap was performed. Surgery was followed by chemotherapy and hormonal therapy where indicated. Dependent on the T status, N status, hormone receptor status, age (≤35 years versus >35 years), and menopausal status, four cycles of Adriamycin/Cyclophosphamide (AC) or six cycles of Cyclophosphamide/Methotrexate/5-FU (CMF) were prescribed for node negative patients, and four cycles of AC followed by four cycles of paclitaxel or, alternatively, three cycles of 5-FU/epirubicin/cyclophosphamide (FEC) followed by three cycles of docetaxel for node positive patients. Endocrine therapy using tamoxifen or aromatase inhibitors was prescribed where indicated. Trastuzumab was added according to the HER2 status and prescribed for at least one year. Triple negative tumor subtype patients were usually treated with four cycles of AC followed by four cycles of paclitaxel. In selected patients neoadjuvant chemotherapy was applied. Postoperative radiotherapy was performed in all patients after BCS. A total dose of 50.4 Gy in 28 fractions was prescribed, followed by a boost of 10 Gy in 5 fractions in all patients younger than 50 years. Postmastectomy radiotherapy of the chest wall was given in patients with at least one positive locoregional lymph node. The prescribed dose was 50 Gy in 25 fractions. Usually opposed tangential beam techniques using three-dimensionally planned conformal radiotherapy (3DCRT) or intensity modulated radiotherapy (IMRT) were applied for the treatment of the whole breast or the chest wall [20]. Follow-up examinations were scheduled every three months in the first year, then every six months for 4 years. PET-CT was performed in many patients during the follow-up. Symptomatic brain metastases were diagnosed by imaging (usually MRI). Breast cancer was classified according to the International Union Against Cancer (UICC), with group clinical and pathological staging according to the American Joint Committee on Cancer (AJCC, 6th edition). Data were entered into a computerized database (MS Access 2010) and analysed using a statistical software package (Statistica 12). This study was approved by the local Institutional Review Board “Institutional Review Board - Saad Specialist Hospital (Registration number: H-05-KH-001, King Abdul-Aziz City of Science and Technology – KACST)” and performed in compliance with the Helsinki Declaration.

### Immunohistochemistry

Sections with a thickness of four μm were cut from paraffin blocks and used for immunohistochemical staining using the iVIEW DAB detection kit on BenchMark autostainer (Ventana, Tucson, AZ, USA). The clones of antibodies SP1, 1E2, and 4B5 were used to evaluate the ER-a, PR, and HER2 status. The Allred scoring system was used to assess the ER and PR status [21]. In summary, a total Allred score was obtained by the summation of proportion score (PS) and intensity score (IS). PS is assigned depending on the proportion of positive cells (0 = none; 1 < 1%; 2 = 1% - < 1/10; 3 = 1/10 - < 1/3; 4 = 1/3 - < 2/3; 5 = 2/3), IS (0 = none; 1 = weak; 2 = intermediate; 3 = strong). A total score of 3 or more was considered as positive; scores 0 and 1 and 2 were considered negative. The American Society of Clinical Oncology/College of American Pathologists (ASCO/CAP) guideline recommendations were used to evaluate the HER2 status [22]. Briefly, score 0 indicates no staining in invasive tumor cells. Score + 1 indicates weak incomplete membrane staining in any proportion of invasive tumor cells or weak complete membrane staining in <10% of cells. Score + 2 indicates complete membrane staining in nonuniform or weak but with obvious circumferential distribution in = 10% of cells, or intense complete membrane staining in = 30% of tumor cells. Score +3 indicates uniform intense membrane staining of >30% of invasive tumor cells. Scores 0 and + 1 were considered negative; + 2 equivocal; and + 3 positive. Gene expression profiling studies have shown that immunohistochemistry of paraffin sections is a reliable surrogate for molecular classification of invasive breast cancers [23-28]. Based on this finding, patients of this study were categorized as follows: luminal A (ER+, PR+, HER2-), luminal B (ER + and/or PR+, HER2+), HER2 overexpressing (ER-, PR-, HER2+), and triple negative (ER-, PR-, HER2-).

### Statistical analysis

The brain metastasis-free survival was estimated using the Kaplan-Meier method, and patient groups were compared using the log rank test. The brain metastasis-free survival was defined as the time between diagnosis of breast cancer and the detection of brain metastases. Patients who have not developed brain metastases were censored at the time of their last follow-up. The simultaneous relationship of multiple prognostic factors on the brain metastasis-free survival was assessed using Cox’s proportional hazard regression analysis. The regression coefficients were estimated by the maximum likelihood method, and model selection was performed by a stepwise strategy using the likelihood ratio test. The Fisher exact test and the Mann-Whitney U test were used to test the difference between patient groups for statistical significance. A 5% significance level was used and all tests are two-sided.

## Results

Three hundred and fifty-two patients were analyzed in this study. The median follow-up time of the censored patients was 19.5 months (3-72 months). Eight patients died during the follow-up. The treatment of the patients consisted of mastectomy in 203 patients (57.7%), breast conserving surgery in 139 patients (39.5%), and non-surgical treatment in 10 patients (2.8%). As expected, compared to what is generally reported in the United States and Europe the patients of this study were diagnosed at a strikingly younger age and more advanced stage of the disease (Table 1) [29]. The median age (minimum-maximum) at diagnosis was 48 years (22-94 years) and the median body mass index (minimum-maximum) 29.9 (17.7-66.4) [30].

**Table 1:** **Univariate analysis** (**Kaplan**-**Meier method**) **of possible factors associated with the development of brain metastasis of non**-**metastatic breast cancer patients at diagnosis**

**Characteristics**		**n**	**%**	**3**-**year brain metastasis**-**free survival**	**-95% CI**	**+95% CI**	**p**-value*
Age (years)							0.10
	≤35	36	10.3	0.87	0.74	1.00	
	>35	316	89.7	0.95	0.92	0.99	
Menopausal status							0.33
	Pre-menopausal	165	47.0	0.95	0.91	0.99	
	Post-menopausal	187	53.0	0.94	0.88	0.99	
Body Mass Index							0.88
	<25	60	17.1	0.95	0.88	1.00	
	25-29	118	33.6	0.96	0.91	1.00	
	≥30	174	49.3	0.93	0.88	0.99	
T stage							0.19
	0-2	200	56.7	0.96	0.92	1.00	
	3-4	152	43.3	0.93	0.87	0.99	
N stage							0.19
	0-1	253	72.1	0.95	0.91	0.99	
	2-3	99	27.9	0.93	0.88	0.99	
Stage							0.10
	Localized	133	37.9	0.95	0.89	1.00	
	Regional	219	62.1	0.94	0.90	0.98	
ER status							<0.001
	Positive	243	69.2	0.99	0.98	1.00	
	Negative	109	30.8	0.84	0.74	0.94	
PR status							0.01
	Positive	216	61.5	0.99	0.96	1.00	
	Negative	136	38.5	0.88	0.80	0.95	
HER2 status							0.31
	Positive	88	25.1	0.98	0.95	1.00	
	Negative	264	74.9	0.93	0.89	0.98	
Tumor subtype							<0.001
	Triple negative	63	17.7	0.78	0.64	0.93	
	Her2 overexpressing	35	10.0	0.96	0.88	1.00	
	Luminal A	201	57.3	0.98	0.95	1.00	
	Luminal B	53	15.1	1.00	1.00	1.00	

*Abbreviation*: 95% *CI* = 95% confidence interval of the 3-year brain metastasis-free survival; * = log rank test.

**Figure 1:**
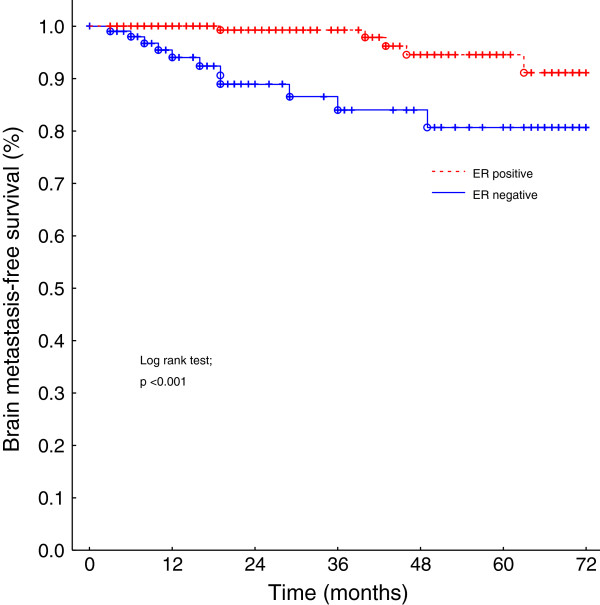
**Brain metastases**-**free survival of breast cancer patients with ER negative versus ER positive tumors.**

**Figure 2:**
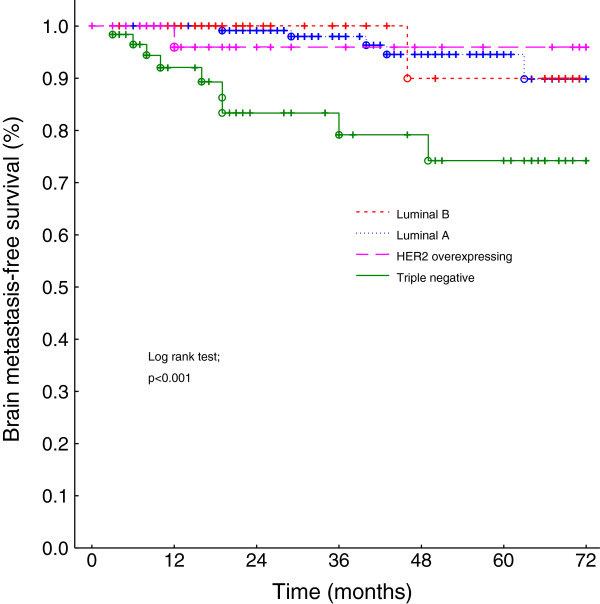
**Brain metastases**-**free survival of breast cancer patients with triple negative versus luminal A, B and HER2 overexpressing.**

On univariate analysis, the ER status (Figure 1), the PR status, and the tumor subtype (Figure 2) had a statistically highly significant impact on the brain metastasis-free survival (Table 1). A closer look at the tumor subtype revealed that the triple negative receptor status had a significantly adverse impact on the brain metastasis-free survival (log rank test, p < 0.01) compared to the combined subtypes luminal A, luminal B and HER2 overexpressing. Young age at diagnosis (≤35 years) and disease stage showed no statistically significant impact (p = 0.10). On multivariate analysis, the only independent significant factor on the brain metastasis-free survival was the ER status (negative ER status, hazard radio (95% confidence interval), 5.1 (1.8-14.6); p = 0.003). Of 109 patients with a negative ER status 11 developed brain metastasis during the follow-up period and of 238 ER positive patients five.

In the subgroup of patients with a minimum follow-up time of 24 months of the censored patients, 16 of 168 patients (9.5%) developed brain metastasis. Of 49 patients with extracranial metastases at first metastatic event 12 (24.4%) later developed brain metastases. The median time interval (minimum-maximum) between the diagnosis of extracranial and brain metastases was 7.5 months (1-30 months). In one patient brain metastases and extracranial metastases were detected at the same time, and three patients developed brain metastases as the first or only distant metastasis. Of 15 patients with extracranial metastases and a negative ER status seven (46.6%) developed brain metastases and of 34 patients with a positive ER status five (14.7%). The difference between the above proportions is statistically significant (Fisher exact test, p = 0.03). The median time interval between the diagnosis of extracranial and brain metastasis of ER negative patients was 5 months (1-11 months) and 18 months (3-30 months) for ER positive patients (Mann-Whitney U test; p = 0.07).

## Discussion

In our retrospective study breast cancer patients with extracranial metastasis and negative ER status exhibited a 46.6% risk of developing brain metastasis during the course of their disease. For this patient group pre-emptive medical intervention such as prophylactic treatment or diagnostic screening may be of benefit.

The most promising pre-emptive medical intervention to improve the outcome may be prophylactic cranial irradiation. Autopsy studies have shown a high frequency of occult brain metastasis in patients with metastatic breast cancer [3,31]. Once brain metastases are diagnosed the survival is usually poor. Reported median survival rates of breast cancer patients with brain metastasis are usually in the range of 3 to 8 months [9,14,16,17,32]. Prophylactic cranial irradiation has been shown to effectively reduce the frequency of brain metastases and to improve the survival in lung cancer [33-35]. In a study of extensive small cell lung cancer, prophylactic cranial irradiation reduced the frequency of brain metastasis from 40.4% to 14.6% (p <0.001) and improved the survival rate from 13.3% to 27.1% one year after randomization [36]. The total radiation dose required for effective prophylactic whole brain irradiation is lower than that required for therapeutic whole brain irradiation of symptomatic brain metastases [37], and the corresponding toxicity is acceptable. Compared to no prophylactic cranial irradiation, prophylactic cranial irradiation showed a negative impact on verbal memory but no or only minimal impact on global cognitive function or global health status [34,35,38]. Due to lack of supporting data prophylactic cranial irradiation has currently no role in breast cancer treatment [32]. The time to the development of brain metastases varies between the patients and it cannot be excluded that in selected patients the seeding of tumors cells in the brain may occur after a prophylactic whole brain irradiation. Future randomized trials are highly desired to evaluate the efficacy of prophylactic cranial irradiation in high risk breast cancer patients.

Another promising prophylactic treatment for patients with HER2 positive disease may be lapatinib, a dual tyrosine-kinase inhibitor of EGFR and HER2. Fewer cases with brain involvement at first progression were observed after treatment with lapatinib in a preliminary analysis of a randomized breast cancer study (4 versus 13, total number of patients 399; p = 0.045) [39]. Lapatinib plus capecitabine has also shown activity as first-line treatment of brain metastases from HER2-positive breast cancer in a phase II study [40].

The value of diagnostic screening for brain metastases of breast cancer patients is unclear. Patients with single metastasis appear to have a significant longer survival than those with multiple metastases [41], and with surgery and stereotactic radiotherapy effective treatment options are available for patients with brain oligometastases. However, early detection of brain metastases has not yet been shown to improve survival [42,43].

The biology underlying the development of brain metastases from breast cancer is only partially understood. The hormone receptor status appears to be associated with the development of brain metastasis as well as with the control of extracranial disease. In addition, it has been shown that the hormonal receptor status is associated with the risk of recurrence of brain metastases after radiosurgery [44]. However, there is a body of evidence suggesting that interactions of metastatic tumour cells with the blood brain barrier and brain microenvironment are also involved in the colonization process [45].

In our study, unfavourable prognostic factors for the brain metastasis-free survival on univariate analysis included negative ER status, negative PR status, triple negative tumor subtype, young age at diagnosis of breast cancer and advanced stage of disease. No high risk group could be defined using these factors for non-metastatic women at diagnosis that would clinically justify pre-emptive medical intervention.

Our results are in good agreement with other reports from the literature. Evans et al. retrospectively analysed 219 breast cancer patients who had died with metastatic disease [17]. The development of brain metastases was significantly related to young age and to a negative ER status. By combining age and ER status (age under 50 years and negative ER status) the authors were able to identify a group of women with a 53% risk of developing brain metastasis. The brain metastases commonly occurred after a good response of liver or lung metastases to chemotherapy, and were often the only site of disease progression. The median time between extracranial metastatic presentation and the development of brain metastases was also very similar in the compared studies (6-9 months; our study, 7.5 months).

Berghoff et al. analysed 213 breast cancer patients with brain metastases [14]. The time interval between the diagnosis of extracranial metastases until diagnosis of brain metastases was significantly different between breast cancer subtypes. Triple negative tumors showed the shortest median time interval (14 months) followed by HER2 positive (18 months) and luminal tumors (34 months). A subgroup analysis showed that patients with a positive ER/HER2 status had a significantly longer time interval compared to ER negative/HER2 positive disease (26 versus 15 months). The authors concluded that patients with triple negative as well as patients with ER negative/HER2 positive disease are at highest risk for developing brain metastases early during their course of disease.

In agreement with our study, a negative hormone receptor status has been identified as significant unfavourable factor for the probability of developing brain metastases in a study of 215 metastatic breast cancer patients [18].

Limitations of our study are related to the retrospective study design and moderate patient numbers. The observed lack of a statistically significant impact of a young age at diagnosis and advanced disease stage on the brain metastasis-free survival (p = 0.10) may be explained by a possible insufficient statistical power of our study. A selection bias cannot be fully excluded and results should be confirmed by future prospective studies.

## Conclusions

In conclusion, breast cancer patients with extracranial metastasis and negative ER status exhibited an almost 50% risk of developing brain metastasis during their course of disease. Future studies are highly desired to evaluate the efficacy of pre-emptive medical intervention such as prophylactic treatment or diagnostic screening for high risk breast cancer patients.

## Abbreviations

BCS: Breast conserving surgery; BMI: Body mass index; EGFR: Epidermal Growth Factor Receptor; ER: Estrogen receptor; HER2: Human epidermal growth factor receptor 2; PR: Progesterone receptor.

## Competing interests

The authors declare that they have no competing interests.

## Authors’ contributions

HS, IB-E, AAN, NA, SA, EF, and VR participated in the acquisition of the data, interpretation of the data and drafting of the manuscript. VR initialized and designed the study and performed the statistical analysis. All authors read and approved the final manuscript.

## Pre-publication history

The pre-publication history for this paper can be accessed here:

http://www.biomedcentral.com/1471-2407/14/289/prepub
